# Respiratory microbes detected in hospitalized adults with acute respiratory infections: associations between influenza A(H1N1)pdm09 virus and intensive care unit admission or fatal outcome in Vietnam (2015–2017)

**DOI:** 10.1186/s12879-021-05988-x

**Published:** 2021-04-06

**Authors:** Phuong Thai Truong, Shinji Saito, Ikuyo Takayama, Hiroyuki Furuya, Binh Gia Nguyen, Thanh Van Do, Phuong Thu Phan, Cuong Duy Do, Co Xuan Dao, Thach The Pham, Tuan Quoc Dang, Chau Quy Ngo, Ngan Thi Le, Vuong Minh Bui, Dung Trung Le, Van Thi Tuong Vu, Thuy Thi Phuong Pham, Takeshi Arashiro, Tsutomu Kageyama, Noriko Nakajima

**Affiliations:** 1 Bach Mai Hospital, Hanoi, Vietnam; 2 Influenza Virus Research Center, National Institute of Infectious Diseases, Tokyo, Japan; 3 Department of Preventive Medicine, Tokai University School of Medicine, Center for Molecular Prevention and Environmental Medicine, Isehara, Japan; 4 NCGM-Bach Mai Hospital Medical Collaboration Center, Hanoi, Vietnam; 5 Department of Pathology, National Institute of Infectious Diseases, Tokyo, Japan

**Keywords:** Respiratory microbes, Acute respiratory infection, Multiplex real-time PCR, Vietnam, A(H1N1)pdm09

## Abstract

**Background:**

Acute respiratory tract infection (ARI) is a leading cause of hospitalization, morbidity, and mortality worldwide. Respiratory microbes that were simultaneously detected in the respiratory tracts of hospitalized adult ARI patients were investigated. Associations between influenza A(H1N1)pdm09 virus (H1N1pdm) detection and intensive care unit (ICU) admission or fatal outcome were determined.

**Methods:**

This prospective observational study was conducted between September 2015 and June 2017 at Bach Mai Hospital, Hanoi, Vietnam. Inclusion criteria were hospitalized patients aged ≥15 years; one or more of symptoms including shortness of breath, sore throat, runny nose, headache, and muscle pain/arthralgia in addition to cough and fever > 37.5 °C; and ≤ 10 days from the onset of symptoms. Twenty-two viruses, 11 bacteria, and one fungus in airway specimens were examined using a commercial multiplex real-time PCR assay. Associations between H1N1pdm detection and ICU admission or fatal outcome were investigated by univariate and multivariate logistic regression analyses.

**Results:**

The total of 269 patients (57.6% male; median age, 51 years) included 69 ICU patients. One or more microbes were detected in the airways of 214 patients (79.6%). Single and multiple microbes were detected in 41.3 and 38.3% of patients, respectively. Influenza A(H3N2) virus was the most frequently detected (35 cases; 13.0%), followed by H1N1pdm (29 cases; 10.8%). Hematological disease was associated with ICU admission (*p* < 0.001) and fatal outcomes (*p* < 0.001) using the corrected significance level (*p* = 0.0033). Sex, age, duration from onset to sampling, or number of detected microbes were not significantly associated with ICU admission or fatal outcomes. H1N1pdm detection was associated with ICU admission (odds ratio [OR] 3.911; 95% confidence interval [CI] 1.671–9.154) and fatal outcome (OR 5.496; 95% CI 1.814–16.653) after adjusting for the confounding factors of comorbidities, bacteria/*Pneumocystis jirovecii* co-detection, and age.

**Conclusions:**

H1N1pdm was associated with severe morbidity and death in adult patients hospitalized with respiratory symptoms. The diagnosis of subtype of influenza virus may be epidemiologically important.

**Supplementary Information:**

The online version contains supplementary material available at 10.1186/s12879-021-05988-x.

## Background

Acute respiratory tract infections (ARIs) include upper respiratory tract infections and lower respiratory tract infections (LRTIs), including pneumonia. ARIs, which are caused by a broad range of microbes, are a leading cause of hospitalization, morbidity, and mortality worldwide [1, 2]. Recent studies examined the etiology of ARI using PCR-based molecular diagnosis techniques that can detect pathogens missed by conventional methods [1–8]. Multiplex real-time PCR (rPCR) assays allow the rapid simultaneous and sensitive detection of multiple viruses, bacteria, and fungi [9, 10]. Thus, multiple microbes are often detected simultaneously in the same clinical specimen. It is important to understand that the PCR-positive microbes are candidate disease-causing pathogens, but not all are necessarily associated with the particular disease [11–14]. For bacteria and fungi in particular, it is difficult to gauge their relevance to the symptoms. Nevertheless, these assays provide epidemiological information that can be useful for treatment planning and prevention of infection.

The proportion of respiratory microbes detected by PCR varies between children and adults, inpatients and outpatients, regions, countries, and during epidemics [15–20]. For hospitalized adult ARI patients, the main causative viruses are influenza A and B viruses, human rhinovirus, human parainfluenza virus, human adenovirus, respiratory syncytial virus, and human metapneumovirus [18–20]. There are three genera of human influenza virus: type A (FluA), type B (FluB), and type C (FluC). Two subtypes of Flu A, A(H1N1)pdm09 virus (H1N1pdm) and A(H3N2) virus (H3), and two lineages of Flu B, B/Yamagata and B/Victoria, are currently co-circulating and have caused human epidemics every year [21]. Several reports involving hospitalized patients suggest that H1N1pdm infection is associated with intensive care unit (ICU) admission or death [22–25].

The primary objective of this study was to investigate 34 respiratory microbes that were simultaneously detected in airway specimens from hospitalized Vietnamese adult ARI patients. The secondary objective was to investigate associations between H1N1pdm detection and ICU admission or fatal outcome.

## Methods

### Study setting

This prospective observational study conducted between September 2015 and June 2017 at Bach Mai Hospital, a 3100 bed tertiary care hospital in Hanoi. It is the biggest government general hospital in Vietnam. Many patients are transferred from hospitals in various provinces of the country to receive more advanced care. The annual inpatient population is approximately 2000 each in the infectious diseases department (78 beds) and respiratory department (123 beds), and 450 in the ICU (73 beds). Patients presenting with severe lower respiratory tract infections, pneumonia, or acute respiratory distress syndrome (ARDS) require mechanical ventilation or extracorporeal membrane oxygenation (ECMO), and they are either admitted or transferred to the ICU.

Patients aged ≥15 years hospitalized in one of the aforementioned departments with one or more presenting symptoms, including shortness of breath, sore throat, runny nose, headache, and muscle pain/arthralgia in addition to cough and fever, were included. The cohort enrolled patients within 10 days of symptom onset, who were either newly hospitalized or those who developed these symptoms during their hospitalization for reasons other than ARI. Patients positive for human immunodeficiency virus (HIV)-1 and those with no available samples within 14 days of onset were excluded.

### Specimens and data collection

Nasopharyngeal swabs (NPS) were collected from all patients. In addition, one sample comprising a throat swab (TS), sputum (SP) sample, or tracheal lavage aspirate (TLA) in case of intubated patients, was collected. The NPS, TS, and SP specimens were added to 1 ml of universal transport medium (Copan, Brescia, Italy), divided into aliquots, and stored along with TLA samples at − 80 °C until use. Demographics, clinical data, and outcomes were collected by retrospective review of patient charts (Table 1).

**Table 1:** Demographic and clinical characteristics of the study participants

Selected variables	All (*n* = 269)	ICU (*n* = 69)	Non-ICU (*n* = 200)	Dead (*n* = 25)	Alive (*n* = 244)
Sex (Male, %)	155 (57.6)	35 (50.7)	120 (60.0)	16 (64.0)	139 (57.0)
Age; Median (IQR); years	51 (33–65)	54 (40–67)	50 (31–64)	56 (47–67)	50 (31–65)
Duration from onset to sampling; Median (IQR); days	5 (3–7)	5 (4–8)	5 (3–7)	6 (4–9)	5 (3–7)
Dead patients	25 (9.3)	23 (33.3)	2 (1.0)		
Comorbidities or conditions	162 (60.2)	45 (65.2)	117 (58.5)	19 (76.0)	143 (58.6)
Lung disease	34 (12.6)	6 (8.7)	28 (14.0)	1 (4.0)	33 (13.5)
Hypertension	27 (10.0)	10 (14.5)	17 (8.5)	3 (12.0)	24 (9.8)
Diabetes mellitus	24 (8.9)	12 (17.4)	12 (6.0)	4 (16.0)	20 (8.2)
Liver disease	24 (8.9)	5 (7.2)	19 (9.5)	4 (16.0)	20 (8.2)
Cardiovascular disease	22 (8.2)	10 (14.5)	12 (6.0)	3 (12.0)	19 (7.8)
Pregnancy	19 (7.1)	3 (4.3)	16 (8.0)	0 (0)	19 (7.8)
Kidney disease	19 (7.1)	6 (8.7)	13 (6.5)	1 (4.0)	18 (7.4)
Collagen disease	11 (4.1)	5 (7.2)	6 (3.0)	3 (12.0)	8 (3.3)
Cancer	9 (3.3)	3 (4.3)	6 (3.0)	0 (0)	9 (3.7)
Tuberculosis	6 (2.2)	2 (2.9)	4 (2.0)	1 (4.0)	5 (2.0)
HBV/HCV	6 (2.2)	1 (1.4)	5 (2.5)	1 (4.0)	5 (2.0)
Hematological disease^*1^	5 (1.9)	5 (7.2)	0 (0)	4 (16.0)	1 (0.4)
Bronchial asthma	4 (1.5)	3 (4.3)	1 (0.5)	0 (0)	4 (1.6)
Immunosuppressive agent usage^*2^	4 (1.5)	4 (5.8)	0 (0)	4 (16.0)	0 (0)
Others	16 (5.9)	4 (5.8)	12 (6.0)	2 (8.0)	14 (5.7)

Data are presented as numbers (%). *IQR* interquartile range. *1: significant differences after Bonferroni correction between ICU and non-ICU patients (Fisher’s exact test, corrected significance level *p* = 0.0033). *1, *2: significant differences after Bonferroni correction between Dead and Alive patients. (Fisher’s exact test, corrected significance level *p* = 0.0033). *ICU* intensive care unit, *HBV/HCV* hepatitis B or hepatitis C

### Multiplex real-time PCR

To collect from as wide an area as possible, two different airway specimens were acquired: NPS (100 μl) and another specimen (TS or SP or TLA; 100 μl). The specimens were mixed and nucleic acids were extracted from the 200 μl mixture using the QIAamp® MinElute Virus Spin kit (Qiagen, Hilden, Germany). Nucleic acids were eluted in 100 μl of RNase-free water and subjected to multiplex rPCR using the Fast Track Diagnostics Respiratory pathogens 33 (FTD33, Fast Track Diagnostics, Esch-sur-Alzette, Luxembourg) plus the CFX96 Real-time PCR Detection System (Bio-Rad, Hercules, CA, USA). In addition, for Flu A-positive patients, subtypes (H1N1pdm or H3) were confirmed using FTD-Flu differentiation (Fast Track Diagnostics). FTD33 combined with FTD-Flu differentiation allowed the detection of 34 respiratory microbes (Supplemental Table). Flu A, H1N1pdm, H3, Flu B, influenza C virus (Flu C), respiratory syncytial virus A/B (RSV A/B), human coronavirus NL63 (NL63), human coronavirus 229E (229E), human coronavirus OC43 (OC43), human coronavirus HKU1 (HKU1), human rhinovirus (HRV), human parainfluenza virus, 1, 2, 3, and 4 (HPIV-1, 2, 3 and 4), human metapneumovirus A/B (HMPV A/B), human bocavirus (HBoV), human adenovirus (HAdV), enterovirus (EV), human parechovirus (HPeV), *Mycoplasma pneumoniae* (*M. pneumoniae*), *Chlamydophila pneumoniae* (*C. pneumoniae*), *Streptococcus pneumoniae* (*S. pneumoniae*), *Klebsiella pneumoniae/Klebsiella variicola* (*K. pneumoniae/K. variicola*), *Haemophilus influenzae* (*H. influenzae*), *Haemophilus influenzae type B* (*H. influenzae B*), *Staphylococcus aureus* (*S. aureus*), *Salmonella species* (*Salmonella. spp.*), *Moraxella catarrhalis* (*M. catarrhalis*), *Legionella pneumophila/longbeachae* (*L. pneumophila/L. longbeachae*), *Pneumocystis jirovecii* (*P. jirovecii*), and *Bordetella species* (*Bordetella. spp.*)This assay used Equine arteritis virus as an internal control. The internal control was added directly to the lysis buffer during each extraction step.

### Statistical analysis

To identify the association between outcomes (ICU admission or fatal outcome) and microbes and other host factors, the univariate associations between the two independent groups were analyzed using the Chi-square test or Fisher’s exact test (categorical variables) and the Mann*-*Whitney U test (continuous variables). Multiple comparisons were made after Bonferroni correction to obtain adjusted *p*-values.

Multivariate logistic regression analysis was used to identify associations between H1N1pdm detection and ICU admission or fatal outcomes after adjusting for age, comorbidities, and bacterial/*P. jirovecii* co-infection. These independent variables were selected based on the reports that comorbidities, age, and secondary bacterial or fungal superinfection are risk factors associated with the severity of influenza [26–28]. Odds ratios (ORs) and 95% confidence intervals (CIs) for each factor were derived from logistic regression analysis. The goodness of fit of the multivariate logistic regression model was determined using the Hosmer-Lemeshow test. All statistical analyses were performed using IBM SPSS statistics for Windows (Version 26.0 J; IBM Corp., Chicago, IL, USA). The level of significance was set at *p* < 0.05, except when Bonferroni correction was applied.

## Results

### Patient characteristics

A total of 269 patients (155 males) patients fulfilled the enrollment criteria. Patients included 69 ICU patients and 200 non-ICU patients (114 in the respiratory department and 86 in the infectious diseases department). Of the 269 patients, 29 patients (including one ICU patient) were hospitalized for reasons other than ARI, but developed ARI after 48 h of hospitalization, and all of these patients were discharged in good health. Demographic and clinical data are summarized in Table 1. The age of the patients ranged from 15 to 94 years. No significant differences were found in median age between ICU and non-ICU patients (*p* = 0.088), or between patients who died and those who survived (*p* = 0.092). There was no significant difference in duration from onset to sampling day between ICU and non-ICU patients (*p* = 0.480) and between dead and alive patients (*p* = 0.585) using the Mann-Whitney U test. Most (23/25, 92%) of the deceased patients were treated in the ICU. The most common comorbidities were lung disease (34 patients), followed by hypertension, diabetes mellitus, liver disease, and cardiovascular disease. Nineteen pregnant women were included. Univariate associations between the two independent groups (each comorbidity vs ICU admission or fatal outcome) were examined. The Bonferroni-corrected significance level was set at 0.05/15 = 0.0033. Diabetes mellitus (Chi-square test, *p =* 0.007), cardiovascular disease (Chi-square test, *p =* 0.039), hematological disease (Fisher’s exact test, *p* < 0.0033), and use of immunosuppressive agents (Fisher’s exact test, *p* = 0.004) were possibly related to ICU admission. Hematological disease and use of immunosuppressive agents were significantly associated with a fatal outcome (Fisher’s exact test, *p* < 0.001 and *p* < 0.001, respectively).

### Detection of single or multiple viruses and bacteria/*P. jirovecii*

One or more microbes were detected in samples from the airways of 214 of 269 patients (79.6%) (Table 2). Of the 34 microbial genomes detectable by FTD33 and FTD-Flu, five (Flu C, EV, HPeV, *C. pneumoniae* and *L. pneumophila/L. longbeacha*) were not detected in any samples. Viruses and bacteria/*P. jirovecii* were detected in 50.9 and 56.1% of the participants, respectively. Single and multiple microbes were detected in 111 (41.3%) and 103 (38.3%) of the patients, respectively. Both virus and bacteria/*P. jirovecii* were detected in 72% (74/103) of patients in whom multiple microbes were detected (Table 2). Among “Microbes detection”, “Virus detection” and “bacteria/*P. jirovecii detection*” in Table 2, only “bacteria/*P. jirovecii* detection” was significantly related to ICU admission (Chi-square test, *p* = 0.009, Bonferroni correction significance level = 0.0167).

**Table 2:** Detection of single or multiple microbes

	Total (*n* = 269)	ICU (*n* = 69)	Non-ICU (*n* = 200)	Dead (*n* = 25)	Alive (*n* = 244)
Microbe detection	214 (79.6%)	60 (86.9%)	154 (77.0%)	21 (84%)	193 (79.1%)
Single microbes	111 (41.3%)	31 (44.9%)	80 (40.0%)	11 (44%)	100 (41.0%)
Multiple microbes	103 (38.3%)	29 (42.0%)	74 (37.0%)	10 (40%)	93 (38.1%)
Virus detection	137 (50.9%)	35 (50.7%)	102 (51.0%)	15 (60%)	122 (50%)
Single virus	55 (20.4%)	11 (15.9%)	44 (22.0%)	5 (20%)	50 (20.5%)
-Multiple viruses	8 (2.97%)	1 (1.45%)	7 (3.50%)	0 (0%)	8 (3.3%)
Both viruses and bacteria/*P. jirovecii*	74 (27.5%)	23 (33.3%)	51 (25.5%)	10 (40%)	64 (26.2%)
Bacteria/*P. jirovecii* detection*	151 (56.1%)	48 (69.5%)	103 (51.5%)	16 (64%)	135 (55.3%)
Single bacteria/*P. jirovecii*	56 (20.8%)	20 (29.0%)	36 (18.0%)	6 (24%)	50 (20.5%)
Multiple bacteria/*P. jirovecii*	21 (7.81%)	5 (7.20%)	16 (8.00%)	0 (0%)	21 (8.6%)
Both viruses and bacteria/*P. jirovecii*	74 (27.5%)	23 (33.3%)	51 (25.5%)	10 (40%)	64 (26.2%)

Data are presented as patient number (%), *: significant differences after Bonferroni correction between ICU and non-ICU patients (Chi-square test, corrected significance level *p* = 0.0167). *ICU* intensive care unit, *P. jirovecii Pneumocystis jirovecii*

The detected microbes and their frequencies are shown in Table 3. H3, H1N1pdm, *K. pneumoniae/K. variicola*, *S. aureus*, and *H. influenzae* were detected in more than 10% of patients. The combinations of co-detected microbes and each frequency are shown in the Supplemental Figure. The percentage of single detection of each microbe, except for HPIV-4, *Salmonella spp*., and *Bordetella. spp.*, were ≤ 50%.

**Table 3:** Detected viruses and bacteria/*P. jirovecii*

Detected microbes	Total (*n* = 269)	ICU (*n* = 69)	non-ICU (*n* = 200)	Dead (*n* = 25)	Alive (*n* = 244)
Viruses					
H3	35 (13.0%)	0	35	0	35
H1N1pdm^*^	29 (10.8%)	13	16	7	22
HRV	24 (8.92%)	5	19	2	22
229E	17 (6.32%)	4	13	1	16
HAdV	14 (5.20%)	5	9	3	11
Flu B	9 (3.35%)	5	4	0	9
HMPV A/B	7 (2.60%)	1	6	1	6
RSV A/B	4 (1.49%)	0	4	0	4
OC43	4 (1.49%)	3	1	2	2
NL63	3 (1.12%)	0	3	0	3
HKU1	3 (1.12%)	1	2	1	2
HPIV-1	3 (1.12%)	0	3	0	3
HBoV	3 (1.12%)	1	2	1	2
HPIV-2	2 (0.07%)	1	1	1	1
HPIV-3	1(0.037%)	0	1	0	1
HPIV-4	1 (0.037%)	1	0	0	1
Bacteria/*P. jirovecii*					
*K. pneumoniae/ K variicola*	73 (27.1%)	26	47	10	63
*S. aureus*	37 (13.8%)	8	29	1	36
*H. influenzae*	30 (11.2%)	12	18	2	28
*S. pneumoniae*	25 (9.29%)	4	21	1	24
*P. jirovecii*	22 (8.18%)	9	13	6	16
*M. catarrhalis*	11 (4.09%)	5	6	1	10
*M. pneumoniae*	8 (2.97%)	0	8	0	8
*H. influenza B*	4 (1.49%)	0	4	0	4
*Salmonella spp.*	3 (1.12%)	0	3	0	3
*Bordetella spp.*	1 (0.037%)	1	0	0	1

*: significant differences between ICU and non-ICU patients (Chi-square test, *p* < 0.05) and significant differences between Dead and Alive patients (Fisher’s exact test, *p* < 0.05). *ICU* intensive care unit, *H3* influenza A/H3N2 virus, *H1N1pdm* influenza A/H1N1pdm virus, *HRV* human rhinovirus, *229E* human coronavirus 229E, *HAdV* human adenovirus, *FluB* influenza B virus, *HMPV A/B* human metapneumovirus A/B, *RSV A/B* respiratory syncytial virus A/B, *OC43* human coronavirus OC43, *NL63* human coronavirus NL63, *HKU1* human coronavirus HKU1, *HPIV-1, 2, 3, and 4* human parainfluenza virus 1, 2, 3, and 4, *HBoV* human bocavirus, *K. pneumoniae/K. variicola Klebsiella pneumoniae/Klebsiella variicola*, S. *aureus Staphylococcus aureus*, *H. influenza Haemophilus influenza*, *S. pneumoniae Streptococcus pneumoniae*, *P. jirovecii Pneumocystis jirovecii*, *M. catarrhalis Moraxella catarrhalis*, *M. pneumoniae Mycoplasma pneumoniae*, *H. influenza B Haemophilus influenzae type B*, *Salmonella spp. Salmonella species*, *Bordetella spp. Bordetella species*

### H1N1pdm detection associated with ICU admission or fatal outcome

Sixty four of 269 enrolled patients (24%) were positive for Flu A. Thirteen of 69 ICU patients (19%) and seven of 25 deceased patients (28%) were positive for H1N1pdm. No ICU or deceased patients were H3 positive (Table 3). Thus, we focused on H1N1pdm and investigated the associations between H1N1pdm detection and ICU admission or fatal outcome. Six patients (including one in the ICU with H1N1pdm) were considered to have nosocomial infections (two patients with H1N1pdm and four patients with H3). Univariate analysis revealed a significant difference in H1N1pdm detection (Chi-square test, *p* = 0.012, significance level *p* = 0.05) between ICU and non-ICU patients, and a significant difference in H1N1pdm detection (Chi-square test, *p* = 0.01, significance level *p* = 0.05) between dead and alive patients (Table 3). We further analyzed the associations between H1N1pdm detection and ICU admission or fatal outcome by multivariate logistic analysis adjusting for confounding factors of presence of comorbidities, bacteria/*P. jirovecii* co-detection, and age. The analysis revealed that H1N1pdm detection was significantly associated with ICU admission (OR 3.911; 95%CI 1.671–9.154; Table 4). The goodness of fit of this model was verified by the Hosmer-Lemeshow test (*p* = 0.720). There was a significant association between H1N1pdm detection and fatal outcome (OR 5.496; 95%CI 1.814–16.653; Table 4). The goodness of fit for this model was also verified by the Hosmer-Lemeshow test (*p* = 0.351).

**Table 4:** Logistic regression analysis of the independent factor prognostic for ICU admission or fatal outcome

	ICU admission			Fatal outcome		
Factor	OR	95% CI	*P* value	OR	95% CI	*P* value
H1N1pdm detection	3.911	1.671–9.154	0.002	5.496	1.814–16.653	0.003
Comorbidities	1.031	0.552–1.923	0.925	1.713	0.623–4.706	0.297
Bac/*P. jirovecii* co-detection	2.823	1.530–5.209	0.001	2.481	0.951–6.473	0.063
Age	1.012	0.996–1.028	0.150	1.013	0.989–1.038	0.299
Constant	0.104	–	0.000	0.019	–	0.000

*OR* odds ratio, *95% CI* 95%confidence interval, *H1N1pdm* influenza A(H1N1)pdm09 virus, *P. jirovecii Pneumocystis jirovecii*

## Discussion

This study enrolled 269 hospitalized adult ARI patients. Twenty-two viruses, 11 bacteria, and one fungus (*P. jirovecii*) in respiratory specimens were examined using a commercial multiplex rPCR assay. Specimens were collected from the nasal cavity and pharynx or trachea. The use of multiplex rPCR allows the rapid and sensitive detection of potential pathogens that are the major causes of symptoms. The assay also screens for other pathogens and can prevent misdiagnosis, even during an epidemic caused by one specific pathogen.

Several reports documented the viral etiology of respiratory infections in hospitalized children and/or adults using multiplex rPCR [15–17]. The virus detection rate (50.9%) in this study is similar to that in a previous report of adults (58.5%) [20], but is lower than that reported in pediatric studies (70–82%) [16, 17]. One possible reason for the difference is that adults already have antibodies against various viruses. In the pediatric studies, the most commonly detected virus varied according to country and ongoing epidemic during the study period. Several adult studies (including this study) most frequently detected influenza virus. The detection could be affected by whether the flu season in the study period occurred during an epidemic [12, 18–20]. Influenza viruses mutate very quickly, so adults who have been infected in the past can be infected again and are at risk of hospitalization [21].

The rate of bacteria/*P. jirovecii* detection (56.1%) was higher than that of viruses, even though the number of screened bacteria/*P. jirovecii* was almost half that of the screened viruses. It is worth remembering that multiplex rPCR also detects the genomes of asymptomatic microbes, persistently infectious viruses, and normal bacterial flora. Therefore, the test does not necessarily signify clinical etiology [11–14]. As an example, a report that analyzed microbes in 312 non-acute specimens from military trainees using the same FTD33 kit showed that viruses and bacteria were present in 13.8 and 93.3% of throat and nasal swabs, respectively, from asymptomatic examinees [11]. It is difficult to verify the clinical significance of the detected microbes using only multiplex rPCR. Etiology should be considered in combination with additional information, such as changes in microbe load and symptoms over time, results of virus isolation or bacterial culture, and changes in serum antibody levels against the microbe. Thus, it is reasonable that detection of multiple microbes in a single patient was not always associated with disease severity (Table 2).

On the other hand, Influenza viruses are not persistent or latent infections, and are rarely detected in asymptomatic controls [11–13]. Several reports have compared disease severity in hospitalized patients with laboratory-confirmed H1N1pdm and H3 infections [22–25, 29, 30]. Focusing on the differences in ICU admission, a recent analysis of a large cohort (696 H1N1pdm and 388 H3 patients from 2010 to 2016) showed that H1N1pdm patients required ICU admission more frequently than those with H3 (*p* < 0.01) [30]. Presently, H1N1pdm detection was significantly associated with both ICU admission and fatal outcome after adjusting for the confounding factors of the presence of comorbidities, bacteria/*P. jirovecii* co-detection, and age. Influenza vaccination coverage is not high in Vietnam. The influenza vaccine prevents severe illness and is reported to be more effective against H1N1pdm infection [31]. If H1N1pdm is associated with severe disease, vaccination should be recommended. Currently, subtyping is not important in a clinical setting, even in ICU-related cases. However, if the attending physician is aware that H1N1pdm can lead to more severe complications (such as ARDS) than H3 and influenza B, the timing of respiratory management and the introduction of anti-inflammatory therapy could be significantly improved. This suggests that subtype diagnosis of influenza may be not only epidemiologically important but also clinically beneficial, although not always mandatory.

This study has several limitations. First, it was a single center study. Second, the number of enrolled patients was small. Third, some control data from asymptomatic adults in Vietnam was unavailable. These adults might harbor bacterial colonization and latent infection. Fourth, the mixing of nasal swabs and lower respiratory tract specimens made it impossible to assess the clinical pathogenicity of LRTI. It might be necessary to take specimens from the alveolar region [32, 33] to more precisely and accurately detect microbes that cause pneumonia. However, sampling of the LRT is very invasive and was therefore was limited to TLA from intubated patients or sputum. Fifth, a patient with a cough and fever, but with a non-infectious respiratory illness, might have been enrolled. Sixth, this was an observational study with limited epidemiological, socioeconomic, and clinical information, and the residual confounders were inevitable limitations. Further studies are needed to establish these associations.

## Conclusions

Microbial genomes were detected simultaneously in the airways of hospitalized ARI patients, and the prevalence of the detected microbes was examined. Sixty-four out of 269 enrolled patients (24%) were found to be positive for Flu A. Subtyping of Flu A-positive patients revealed the association of H1N1pdm with severe morbidity and death in adult patients requiring hospitalization. This finding suggests that the subtyping of influenza virus is epidemiologically important and that H1N1pdm-positive hospitalized patients may potentially be severely ill.

## Supplementary Information

**Additional file 1: Supplemental Figure.** Combination of pathogens in patients with multiple detections. The numbers in gray present the number of patients harboring a single pathogen. The other data do not always represent the number of patients; rather they represent the number of each pathogen that was detected. For example, a patient with simultaneous detection of H1N1pdm, HRV, and *K. pneumoniae/K. variicola* is included in the number of simultaneous detections of H1 and HRV as well as in the number of simultaneous detections of H1 and *K. pneumoniae/K. variicola*. H3, influenza A(H3N2) virus; H1N1pdm, influenza A(H1N1)pdm09 virus; HRV, human rhinovirus; 229E, human coronavirus 229E; HAdV, human adenovirus; FluB, influenza B virus; HMPV A/B, human metapneumovirus A/B; RSV A/B, respiratory syncytial virus A/B; OC43, human coronavirus OC43; NL63, human coronavirus NL63; HKU1, human coronavirus HKU1; HPIV-1, human parainfluenza virus-1; HBoV, human bocavirus; HPIV-2, human parainfluenza viruses-2; HPIV-3, human parainfluenza virus-3; HPIV-4, human parainfluenza virus-4; *K. pneumoniae/K. variicola*, *Klebsiella pneumoniae/Klebsiella variicola*; *S. aureus*, *Staphylococcus aureus*; *H. influenzae*, *Haemophilus influenza*; *S. pneumoniae*, *Streptococcus pneumoniae*; *P. jirovecii*, *Pneumocystis jirovecii*; *M. catarrhalis*, *Moraxella catarrhalis*; *M. pneumoniae*, *Mycoplasma pneumoniae*; *H. influenzae B*, *Haemophilus influenzae type B*; *Salmonella. spp.*, *Salmonella species*; *Bordetella spp.*, *Bordetella species*.

**Additional file 2: Supplement Table.** Microbes detected by FTD33 / FTD-Flu differentiation.

## Data Availability

The datasets used and/or analyzed during the current study are available from the corresponding author upon reasonable request.

## Abbreviations

ARI: Acute respiratory tract infection
rPCR: real-time PCR
Flu A: Influenza A virus
H1N1pdm: Influenza A(H1N1)pdm virus
H3: Influenza A(H3N2) virus
Flu B: Influenza B virus
ICU: Intensive care unit
NPS: Nasopharyngeal swab
TS: Throat swab
SP: Sputum
TLA: Tracheal lavage aspirate
FTD33: Fast track diagnostics respiratory pathogens 33 kit
FTD-Flu: Fast track diagnostics flu differentiation kit
Flu C: Influenza C virus
RSV A/B: Respiratory syncytial virus A/B
NL63: Human coronavirus NL63
229E: Human coronavirus 229E
OC43: Human coronavirus OC43
HKU1: Human coronaviruses HKU1
HRV: Human rhinovirus
HPIV-1, 2, 3, and 4: Human parainfluenza virus 1, 2, 3, and 4
HMPV A/B: Human metapneumovirus A/B
HBoV: Human bocavirus
HAdV: Human adenovirus
EV: Enterovirus
HPeV: Human parechovirus
*M. pneumoniae*: *Mycoplasma pneumoniae*
*C. pneumoniae*: *Chlamydophila pneumoniae*
*S. pneumoniae*: *Streptococcus pneumoniae*
*K. pneumoniae/K. variicola*: *Klebsiella pneumoniae/Klebsiella variicola*
*H. influenzae*: *Haemophilus influenzae*
*H. influenzae B*: *Haemophilus influenzae type B*
*S. aureus*: *Staphylococcus aureus*
*Salmonella. spp.*: *Salmonella species*
*M. catarrhalis*: *Moraxella catarrhalis*
*L. pneumoniae/ L. longbeachae*: *Legionella pneumophila/ Legionella longbeachae*
*P. jirovecii*: *Pneumocystis jirovecii*
*Bordetella. Species*: *Bordetella spp.*
ARDS: Acute respiratory distress syndrome
