# Supplementary figures and images for: Respiratory microbes detected in hospitalized adults with acute respiratory infections: associations between influenza A(H1N1)pdm09 virus and intensive care unit admission or fatal outcome in Vietnam (2015–2017)
Source: BMC Infect Dis. 2021 Apr 6;21:320. doi: 10.1186/s12879-021-05988-x (PMC8023524; doi:10.1186/s12879-021-05988-x)

## Slide 1
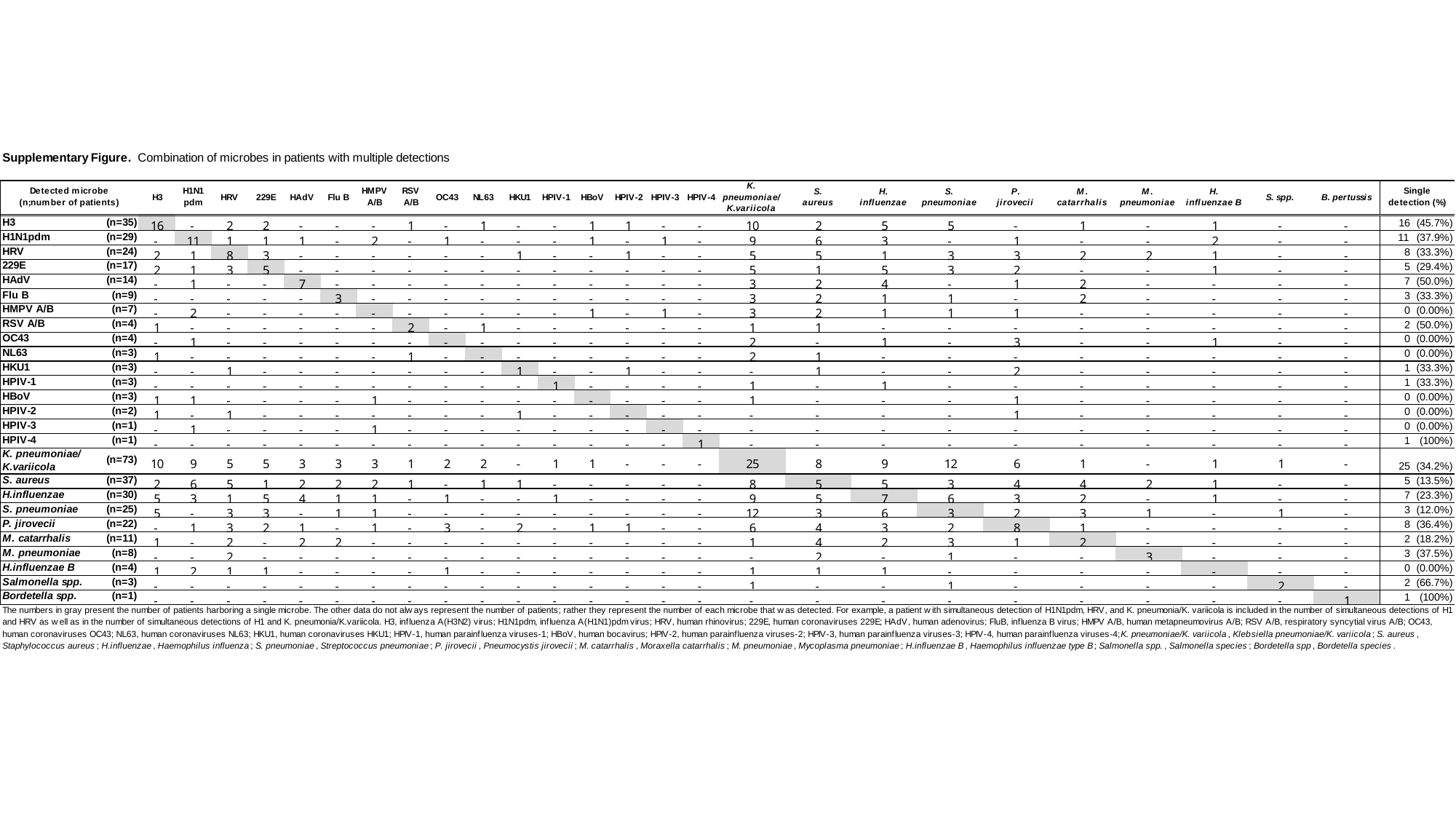

Supplement: Supplementary file 1 — Additional file 1: Supplemental Figure. Combination of pathogens in patients with multiple detections. The numbers in gray present the number of patients harboring a single pathogen. The other data do not always represent the number of patients; rather they represent the number of each pathogen that was detected. For example, a patient with simultaneous detection of H1N1pdm, HRV, and K. pneumoniae/K. variicola is included in the number of simultaneous detections of H1 and HRV as well as in the number of simultaneous detections of H1 and K. pneumoniae/K. variicola. H3, influenza A(H3N2) virus; H1N1pdm, influenza A(H1N1)pdm09 virus; HRV, human rhinovirus; 229E, human coronavirus 229E; HAdV, human adenovirus; FluB, influenza B virus; HMPV A/B, human metapneumovirus A/B; RSV A/B, respiratory syncytial virus A/B; OC43, human coronavirus OC43; NL63, human coronavirus NL63; HKU1, human coronavirus HKU1; HPIV-1, human parainfluenza virus-1; HBoV, human bocavirus; HPIV-2, human parainfluenza viruses-2; HPIV-3, human parainfluenza virus-3; HPIV-4, human parainfluenza virus-4; K. pneumoniae/K. variicola, Klebsiella pneumoniae/Klebsiella variicola; S. aureus, Staphylococcus aureus; H. influenzae, Haemophilus influenza; S. pneumoniae, Streptococcus pneumoniae; P. jirovecii, Pneumocystis jirovecii; M. catarrhalis, Moraxella catarrhalis; M. pneumoniae, Mycoplasma pneumoniae; H. influenzae B, Haemophilus influenzae type B; Salmonella. spp., Salmonella species; Bordetella spp., Bordetella species. [file 12879_2021_5988_MOESM1_ESM.pptx]
