# Supplementary material for: Respiratory microbes detected in hospitalized adults with acute respiratory infections: associations between influenza A(H1N1)pdm09 virus and intensive care unit admission or fatal outcome in Vietnam (2015–2017)
Source: BMC Infect Dis. 2021 Apr 6;21:320. doi: 10.1186/s12879-021-05988-x (PMC8023524; doi:10.1186/s12879-021-05988-x)
Supplement: Supplementary file 2 — Additional file 2: Supplement Table. Microbes detected by FTD33 / FTD-Flu differentiation. [file 12879_2021_5988_MOESM2_ESM.docx]

| Detected microbes | Abbreviation |
| --- | --- |
| influenza A virus | FluA |
| influenza A(H3N2) virus | H3 |
| influenza A(H1N1)pdm09 virus | H1N1pdm |
| influenza B virus | FluB |
| influenza C virus | FluC |
| respiratory syncytial virus A/B | RSV A/B |
| human coronavirus NL63 | NL63 |
| human coronavirus 229E | 229E |
| human coronavirus OC43 | OC43 |
| human coronavirus HKU1 | HKU1 |
| human rhinovirus | HRV |
| human parainfluenza virus 1 | HPIV-1 |
| human parainfluenza virus 2 | HPIV-2 |
| human parainfluenza virus 3 | HPIV-3 |
| human parainfluenza virus 4 | HPIV-4 |
| human metapneumovirus A/B | HMVPV A/B |
| human bocavirus | HBoV |
| human adenovirus | HAdV |
| enterovirus | EV |
| human parechovirus | HPeV |
| *Mycoplasma pneumoniae* | *M. pneumoniae* |
| *Chlamydophila pneumoniae* | *C. pneumoniae* |
| *Streptococcus pneumoniae* | *S. pneumoniae* |
| *Klebsiella pneumoniae/Klebsiella variicola* | *K. pneumoniae/K. variicola* |
| *Haemophilus influenzae* | *H. influenzae* |
| *Haemophilus influenzae type B* | *H. influenzae B* |
| *Staphylococcus aureus* | *S. aureus* |
| *Salmonella species* | *Salmonella. spp.* |
| *Moraxella catarrhalis* | *M. catarrhalis* |
| *Legionella pneumophila/Legionella longbeachae* | *L. pneumophila/L. longbeachae* |
| *Pneumocystis jirovecii* | *P. jirovecii* |
| *Bordetella species* | *Bordetella. spp.* |

Supplement Table

Microbes detected by FTD33 / FTD-Flu differentiation
